# Postoperative spindle cell nodule of the bladder: A case report and review of the literature

**DOI:** 10.3892/ol.2014.1927

**Published:** 2014-02-28

**Authors:** JIYU ZHAO, HAO PING, NIANZENG XING

**Affiliations:** Department of Urology, Beijing Chaoyang Hospital, Capital Medical University, Beijing 100020, P.R. China

**Keywords:** postoperative spindle cell nodule, bladder cancer, misdiagnosis

## Abstract

Postoperative spindle cell nodule (PSCN) of the bladder is a rare condition. It is a type of benign lesion frequently misdiagnosed as sarcomatoid carcinoma on the basis of similar cell morphology. The present report describes a tumor affecting a 71-year-old male who had undergone three transurethral resections. Pathological results suggested a diagnosis of sarcomatoid carcinoma, and therefore a radical cystectomy was performed. However, the tumor was later identified as a PSCN. In order to prevent such misdiagnosis, this study reviews relevant articles concerning postoperative spindle cell nodules of the bladder and compares PSCN and sarcomatoid carcinomas to identify specific characteristics of PSCN. Finally, the report emphasizes the importance of careful pathological examination in rare cases such as PSCN and sarcomatoid carcinoma.

## Introduction

Postoperative spindle cell nodule (PSCN) of the bladder is a rare non-neoplastic lesion of the bladder consisting of a reactive proliferation of spindle cells. PSCN occurs between several weeks or months following surgery at the site of surgical intervention, such as transurethral resection or biopsy (1). Although PSCN resembles a sarcoma, it has a favorable prognosis and conservative management is considered a reasonable treatment option for the disease (2). The majority of the tumors of the bladder are malignant, with transitional cell carcinoma being the most common type of malignant tumor. However, other tumors may lead to misdiagnosis. This report presents a case of PSCN of the bladder and reviews the literature to summarize its characteristics and assist in avoiding misdiagnosis and unnecessary treatment. Patient provided written informed consent.

## Case report

**Figure 1:**
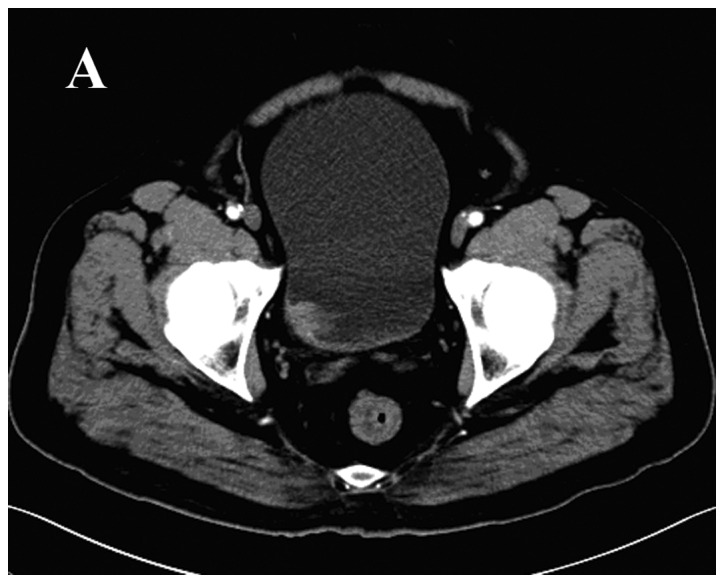

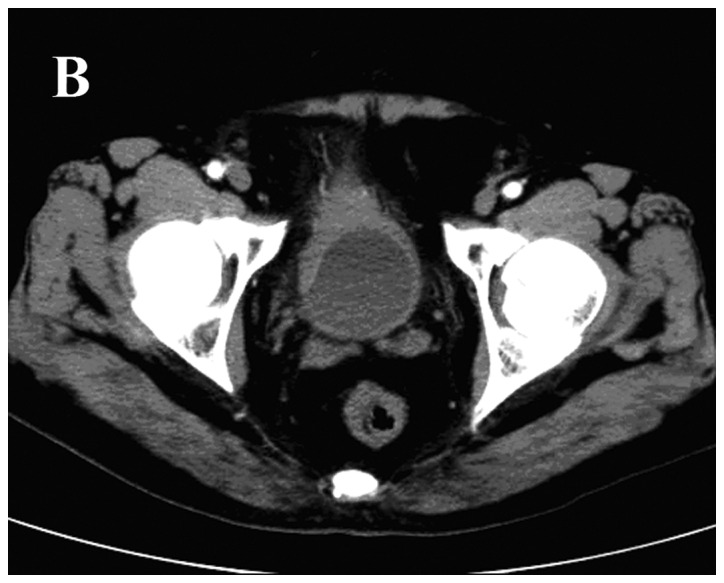
Radiological features of the case. Arterial-phase computed tomography scan revealing (A) a 2.5 cm-diameter solid pedunculated mass at the right side of the bladder wall, and (B) thickened anterior and right bladder walls with significant mucous membrane enhancement. No significant bladder mass was detected.

A 71-year-old male patient underwent cystoscopy on 6th February 2011 for painless, total gross hematuria. A 2.5 cm-diameter, solid, pedunculated mass with a necrotic lesion on the surface of the right side of the bladder wall was identified (Fig. 1A). The patient had a 10-year history of hypertension and had received a coronary artery stent for coronary heart disease five years previously, in addition to a four-year history of gout. Bladder mass biopsy revealed low-grade papillary urothelial carcinoma. The patient underwent transurethral resection (TUR) of the lesion. Five weeks later, the patient underwent a second TUR for persistent gross hematuria. A mass was identified on the right-side wall, and the pathological results were compatible with a diagnosis of sarcomatoid carcinoma. Seven months later, the patient underwent a third TUR for tumor recurrence. The histopathological findings suggested a diagnosis of sarcomatoid carcinoma, but no bladder mass was detected by computed tomography scan prior to the resection (Fig. 1B). In accordance with the pathological results, a radical cystectomy with regional lymph node dissection and Bricker ileal conduit urinary diversion was performed. The final pathological sections indicated a diagnosis of PSCN.

**Figure 2:**
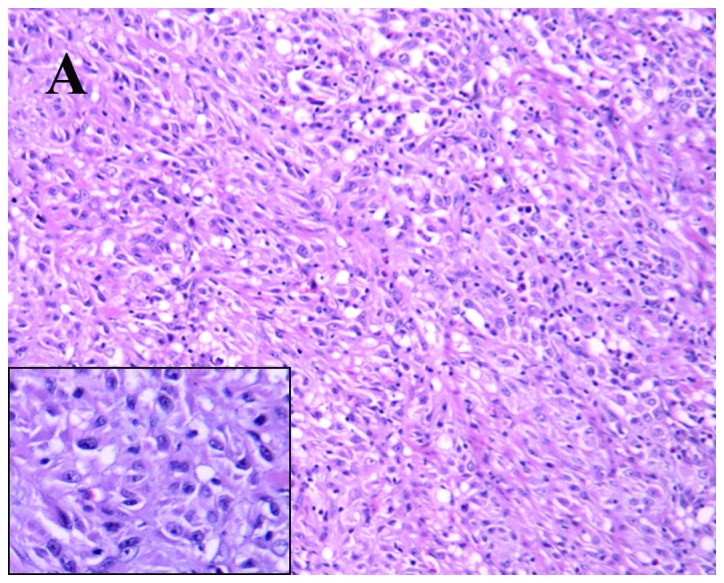

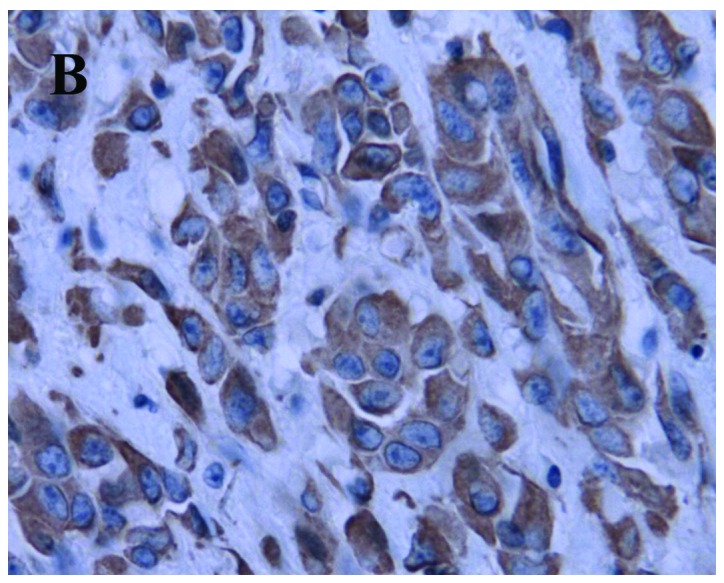

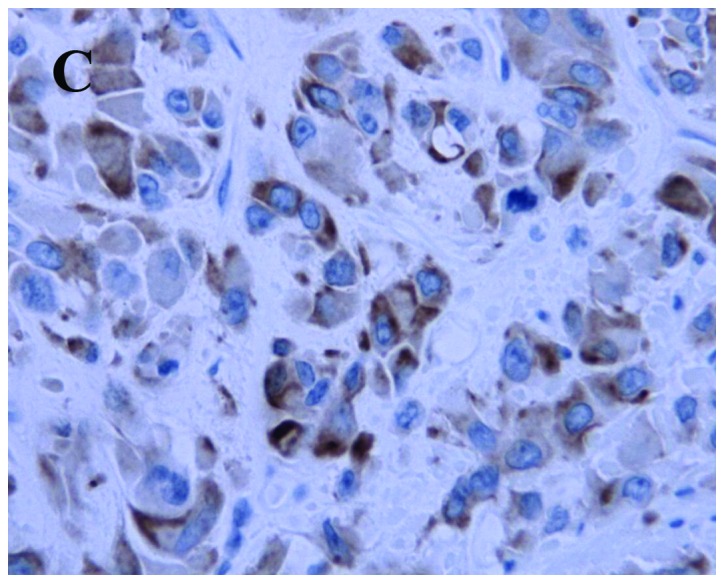

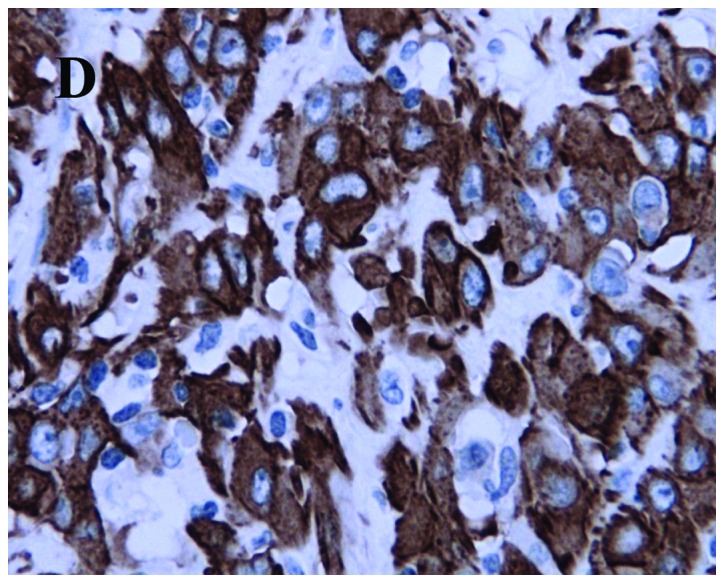
Histopathological and immunohistochemical features of the case: (A) Irregular fascicles of bland-looking spindle cells with several fascicles of chronic inflammatory cells scattered in myxoid stroma (hematoxylin and eosin staining; magnification, ×200); (B) vimentin is strongly expressed in the spindle cells (magnification, ×400); (C) positive cytokeratin immunohistochemical staining (magnification, ×400); and (D) focal positivity of smooth muscle actin staining (magnification, ×400).

Immunostaining was performed on fixed sections embedded in paraffin with appropriate controls. Stains for the expression of vimentin and cytokeratin were positive, and focal positivity for smooth muscle actin (SMA) and cytokeratin (CK)8/18 was noted (Fig. 2). The Ki-67 index was 15%. Immunostaining for desmin, epithelial membrane antigen (EMA), S-100, CK34β E12, CK14, P63, CK7 and CK20 was negative. The patient received no further therapy and was without evidence of disease 14 months later.

## Discussion

PSCN of the urinary bladder was first reported in 1984 by Proppe *et al* (3), who coined the term ‘post-operative spindle cell nodule’ in a study including a series of eight patients with proliferation of spindle cells following surgical procedures. In the article, Proppe reported in detail the clinical findings, pathological features and initial diagnoses of these cases. However, due to the limitations of the technology at the time, the authors did not analyze the lesions using immunohistochemical methods and examined the nodules using only light microscopy.

Microscopic examination revealed several important characteristics of PSCN (4,5), including intersecting fascicles of spindle cells, small blood vessels and various chronic inflammatory cells scattered in myxoid stroma. The spindle cells were arranged in bundles or nodules, which had compacted acidophilic cytoplasm and elongated, blunt-ended nuclei. There were numerous mitotic figures among the spindle cells but without significant atypia. The spindle cells frequently invaded the bladder walls between smooth muscles and penetrated through the walls into surrounding soft tissue without disrupting the muscle fibers (2). Small foci of hemorrhage and moderate edema were present in the stroma. The inflammatory cells included plasma cells, lymphocytes and macrophages and, in a few cases (5), neutrophils and eosinophils were identified during examination. Necrosis and calcification were absent.

PSCN and sarcomatoid carcinoma are difficult to distinguish from one another (2,6). The two diseases share numerous similarities, including numerous spindle cells scattered in myxoid stroma and various chronic inflammatory cells in the surroundings. However, sarcomatoid carcinoma is a rare malignancy of the bladder with markedly atypical spindle cell proliferation and increased irregular mitoses.

PubMed and Embase were searched for all reports of spindle cell bladder tumors. The inclusion criterion for cases was a diagnosis of PSCN of the bladder by the referring pathologist. Six articles were identified, comprising 21 cases, including the patient of the present report. General patient information and histological data were tabulated (Tables I and II). Patients ranged in age between 40 and 85 years (mean, 65 years). Males were affected more than females (1.6:1). The majority of the patients presented with hematuria (4/6). The lesions ranged in size between 0.4 and 4.5 cm (mean*,* 2.0 cm). The percentage of cases without invasion of the muscularis was 62.5% (10/16) and, immunohistochemically, the lesional cells of PSCN stained positive for cluster of differentiation 68 (100%), vimentin (100%), CK AE1/AE3 (84%), SMA (81%), muscle-specific actin (MSA; 80%), desmin (57%), p53 (60%) and EMA (14%). S-100 protein was negative. Micci *et al* (7) identified three signals for chromosome 7 in one case by interphase fluorescence *in situ* hybridization. Another report demonstrated that PSCN and sarcomatoid carcinoma stained positive for vimentin, and that some PSCNs stained positive for CK and EMA, which may lead to misdiagnosis of sarcomatoid carcinomas (6). However, MSA and SMA are negative in sarcomatoid carcinoma tissue but positive in PSCN tissue. Differences are also apparent under electron microscopy, which reveals fibroblastic or myofibroblastic differentiation in PSCN in contrast to epithelial differentiation in sarcomatoid carcinoma (8,9).

The majority of PSCNs of the bladder were managed locally by TUR. Partial cystectomies were performed on two patients, and a radical cystectomy was performed on the patient of the present study. Whether PSCN was only a type of reactive proliferation or a true neoplasm remains unclear. Follow-up data was available for 20 patients. No tumors recurred or metastasized in 17 patients, suggesting that PSCN tends to be a benign lesion. Two patients succumbed to other diseases. Only one patient was diagnosed with carcinoma *in situ* of the bladder 12 months after surgery, although the association between the recurrence and the PSCN is not yet clear. Due to the good postoperative follow-up results, the best choice for symptomatic patients is likely TUR, while partial cystectomy and radical cystectomy should not be recommended. Bladder-sparing surgery is advised to preserve the patient’s quality of life.

In conclusion, this report has described a patient with a postoperative spindle cell nodule that occurred in the bladder following a TUR for treatment of bladder carcinoma. In occasional cases, the recurrence of bladder mass following surgical procedures may not be a malignant tumor, but a reactive proliferation such as PSCN. Thus, it is necessary to perform a preoperative biopsy before each surgery for diagnosis. Following detailed pathological analysis and clear diagnosis, TUR is the ideal treatment for avoiding extensive surgery.

## Figures and Tables

**Table I:** Clinical data of 21 patients with postoperative spindle cell nodule of the bladder.

Case	Age, years	Gender	Prior bladder procedures	Symptoms	Nodule size, cm	T stage	Therapy	Follow-up status, months	Study
1	49	M	Biopsy, 2 months prior^a^	Hematuria	3.0	2	PC	CIS (12)	Iczkowski *et al* (10)
2	83	M	TUR, 1 week prior^a^	Hematuria	1.7	2	TUR	NET (8.5)	Iczkowski *et al* (10)
3	56	M	7 biopsies, 5 months prior^a^	None	0.8	ND	TUR	NET (30)	Iczkowski *et al* (10)
4	70	M	13 TURs, 13 months prior^a^	None	0.4	ND	TUR	NET (26.4)	Iczkowski *et al* (10)
5	64	M	TUR, 3 months prior^a^	Hematuria	ND	2	TUR	NET (24)	Micci *et al* (7)
6	75	F	TUR	ND	ND	1	TUR	NET (27)	Spiess *et al* (11)
7	49	F	TUR	ND	ND	a	TUR	NET(13)	Spiess *et al* (11)
8	78	F	TUR	ND	ND	a	TUR	NET (2)	Spiess *et al* (11)
9	71	M	TUR	ND	ND	2	PC	NET (67)	Spiess *et al* (11)
10	40	M	TUR	ND	ND	1	TUR	NET (45)	Spiess *et al* (11)
11	85	M	TUR	ND	ND	1	TUR	NET (46)	Spiess *et al* (11)
12	76	F	TUR	ND	ND	1	TUR	DOD (48)	Spiess *et al* (11)
13	62	M	TUR	ND	ND	a	TUR	NET (62)	Spiess *et al* (11)
14	72	F	TUR	ND	ND	1	TUR	DOD (48)	Spiess *et al* (11)
15	66	F	TUR	ND	ND	1	TUR	NET (34)	Spiess *et al* (11)
16	72	M	ND	ND	4.5	<2	ND	NET (62)	Montgomery *et al* (5)
17	73	F	ND	ND	ND	≥3	ND	ND	Montgomery *et al* (5)
18	45	F	TUR, 2 weeks prior^a^	Hematuria	2.0	ND	TUR	NET (24)	Lo *et al* (12)
19	55	M	ND	Hematuria	ND	ND	TUR	NET (12)	Wick *et al* (13)
20	60	M	ND	None	ND	ND	TUR	NET (6)	Wick *et al* (13)
21	71	M	TUR, 5 weeks prior^a^	Hematuria	1.5	3	RC	NET (5)	Present case

a Months prior to the bladder procedures.

TUR, transurethral resection; ND, no data; PC, partial cystectomy; RC, radical cystectomy; CIS, carcinoma *in situ*; NET, no evidence of tumor; DOD, deceased (other disease); a, non-invasive papillary carcinoma.

**Table II:** Immunohistochemical reactivity in 21 cases of postoperative spindle cell nodule of bladder.

Study	p53	CK AE 1/3	EMA	SMA	MSA	Desmin	Vimentin	S-100	CD 68
Iczkowski *et al* (10)	3/4	2/4	1/3	2/4	2/3	2/3	4/4	0/3	ND
Micci *et al* (7)	ND	ND	ND	ND	ND	ND	ND	ND	ND
Spiess *et al* (11)	ND	10/10	ND	10/10	ND	ND	10/10	ND	10/10
Montgomery *et al* (5)	ND	1/1	ND	ND	ND	ND	ND	ND	ND
Lo *et al* (12)	0/1	0/1	0/1	0/1	ND	0/1	1/1	0/1	ND
Wick *et al* (13)	ND	2/2	0/2	ND	2/2	2/2	2/2	0/2	ND
Present case	ND	1/1	0/1	1/1	ND	0/1	1/1	0/1	ND
Total	3/5	16/19	1/7	13/16	4/5	4/7	18/18	0/7	10/10

CK AE 1/3, cytokeratin AE 1/3; EMA, epithelial membrane antigen; SMA, smooth muscle actin; MSA, muscle-specific actin; CD, cluster of differentiation; ND, no data.
